# Enantioseparation, Stereochemical Assignment and Chiral Recognition Mechanism of Sulfoxide-Containing Drugs

**DOI:** 10.3390/molecules23102680

**Published:** 2018-10-18

**Authors:** Fei Xiong, Bei-Bei Yang, Jie Zhang, Li Li

**Affiliations:** Beijing Key Laboratory of Active Substances Discovery and Druggability Evaluation, Institute of Materia Medica, Chinese Academy of Medical Sciences & Peking Union Medical College, Beijing 100050, China; xxf@imm.ac.cn (F.X.); yangbetty@imm.ac.cn (B.-B.Y.); zhd@imm.ac.cn (J.Z.)

**Keywords:** chiral sulfoxide drugs, enantiomer elution order, electronic circular dichroism, quantum chemical calculation, molecular docking

## Abstract

The distinct pharmacodynamic and pharmacokinetic properties of enantiopure sulfoxide drugs have stimulated us to systematically investigate their chiral separation, stereochemical assignment, and chiral recognition mechanism. Herein, four clinically widely-used sulfoxide drugs were chosen and optically resolved on various chiral stationary phases (CSPs). Theoretical simulations including electronic circular dichroism (ECD) calculation and molecular docking were adopted to assign the stereochemistry and reveal the underlying chiral recognition mechanism. Our results showed that the sequence of calculated mean binding energies between each pair of enantiomers and CSP matched exactly with experimentally observed enantiomeric elution order (EEO). It was also found that the length of hydrogen bond might contribute dominantly the interaction between two enantiomers and CSP. We hope our study could provide a fresh perspective to explore the stereochemistry and chiral recognition mechanism of chiral drugs.

## 1. Introduction

Chirality has a profound impact on the chemical processes of biology. Sulfur is an important bio-element, whose certain compounds can exhibit chirality such as sulfoxides, or sulfoximines. Although chiral sulfur compounds have received much less attention than chiral carbon compounds, chiral sulfoxides possessing a pyramidal structure comprise a unique class of drugs containing a chiral heteroatom [1]. The high enantioselectivity of biological events has prompted researchers to develop enantiopure sulfoxide drugs due to their distinct pharmacodynamic and pharmacokinetic properties [2,3]. In addition to its application in medicinal chemistry, sulfoxide is also significant functional group for its use as a ligand and chiral auxiliary in asymmetric synthesis [4,5].

**Figure 1:**
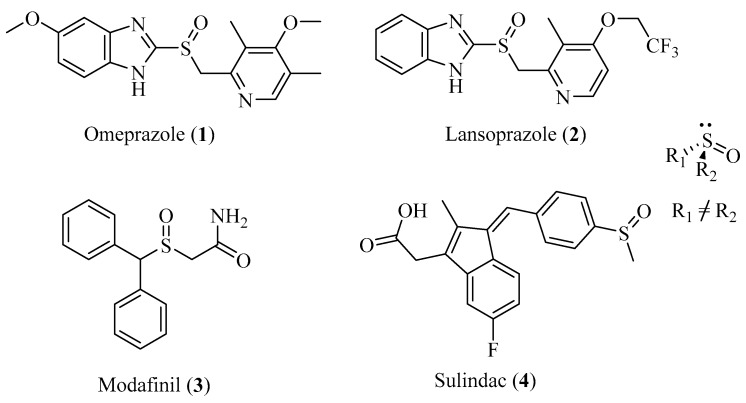
Chemical structures of chiral sulfoxides.

Compounds **1**–**4** (Figure 1) are clinically widely-used sulfoxide-containing drugs, and represent two typical structures of sulfoxides, i.e., aromatic alkyl sulfoxides (**1**, **2**, **4**) and alkyl sulfoxide (**3**). Both **1** and **2** earned enough attention because of marvelous market success, and **3** is a wake-promoting drug used in the war [6,7,8]. Recently, the non-steroidal anti-inflammatory drug **4** and its derivatives aroused the interest of researchers for its potent anticancer effect [9,10].

In the last two decades, hundreds of papers reported the chiral separation methods of sulfoxides including capillary electrophoresis [11,12], chemical resolution [13,14], HPLC [15,16,17,18], and supercritical fluid chromatography [19,20]. Amongst all, we focused on chiral HPLC, which might be a favorable and effective way for both analytical and preparative separations of chiral compounds [21]. There are several HPLC methods using different types of CSPs for **1**, **2**, and 3 [7,16,22,23,24,25,26,27,28,29,30]. Relatively few studies have been reported on the synthesis and chiral enantioseparation of **4** [31,32,33,34,35,36]. Although the piecemeal chiral separation of these compounds was previously reported, a systematic and comprehensive study is beneficial to seek for general clues to the separation of chiral sulfoxide drugs.

Absolute configuration (AC) assignment is crucial for the stereochemical characterization of chiral compounds. For these four drugs, this was previously fulfilled by single-crystal X-ray diffraction, chemical synthesis, or comparison of circular dichroism spectra with their strongly correlatives [7,24,29,34,37,38,39]. However, it might be interesting and useful to establish directly their stereochemistry without reference samples and spectra. Presently, ECD spectra together with quantum chemical calculations using time-dependent density functional theory (TDDFT) have provided a feasible and reliable way to facilitate the AC assignments of chiral drugs and natural products [15,18,40,41,42,43,44]. Hence, TDDFT calculations will be adopted to simulate ECD spectra of sulfoxides in this study.

Recently, rapid development of binding energy computation software has revolutionized the study and elucidation of chiral separation mechanism, and binding energy computation could be applied to reproduce the elution orders of enantiomers [44,45]. In this work, we selected these four sulfoxide drugs to take a systematic experimental and theoretical investigation, for the aim of disclosing some potential regulation during the process of chiral separation and characterization of sulfoxides. Specially, chiral resolution of **1**–**4** will be tested on commercially available and cost-effective Chiralpak AD-H, AS-H, Chiralcel OD-H, and OJ-H chiral columns, followed by AC assignment and chiral separation mechanism analysis.

## 2. Results and Discussion

### 2.1. Chiral Resolution of ***1***–***4***

#### 2.1.1. Factors Affecting Optical Resolution

The influence of the flow rate, mobile phase composition, column temperature, and acidic modifier on resolution was taken into consideration. With the increasing proportion of n-hexane (n-Hex) in the mobile phase, Rs increased in a non-linear manner (Figure S1A). It was manifested that Rs changed slightly with increasing temperature (Figure S1B) and steadily decreased when the flow rate increased from 0.5 to 1.0 mL/min (Figure S1C). Additionally, the column temperature can also affect the protonation of analytes and the CSP, which can then alter the chiral interactions between the solute and the stationary phase.

The great concern about the tailing phenomenon of acid compound **4** can be overcome using an acidic additive like formic acid (FA). Preliminary study showed that resolution of racemic **4** could only be attained on the Chiralpak AD-H column herein, and FA was then added to make a flagrant contrast. Fortunately, FA could achieve better separation of **4** through decreasing tailing, sharpening the peaks, and improving the selectivity (Table S1 and Figure S2). FA in the mobile phase might protonate both **4** and residual -NH- groups on CSP arising from the weak protonation capability of FA. Hence, non-enantioselective association between **4** and the CSP was partly inhibited. Subsequently, it improved the mass transfer, which greatly enhanced the chiral recognition ability of the stationary phase.

#### 2.1.2. The Optimal Resolution and Enantiomeric Elution Order

**Figure 2:**
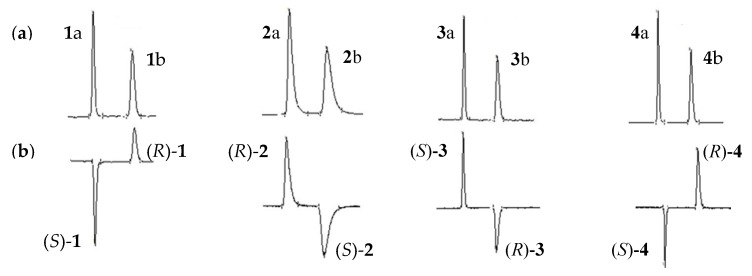
The UV (**a**) and ECD chromatograms (**b**) of racemic **1**–**4** on various columns. **1** on the ChiralPak AD-H column, **2** on the Chiralcel OD-H column, **3** on the ChiralPak AS-H column, and **4** on the ChiralPak AD-H column. Detection wavelengths of **1**–**4** are 275, 275, 240, and 285 nm, respectively.

The separation conditions for compounds **1**–**4** on four chiral columns are listed in Table 1, and the optimal UV and ECD chromatograms are shown in Figure 2 and Figures S3–S6. The more satisfactory results of optimal resolution of racemic **1** on the Chiralpak AD-H column, **2** on the Chiralcel OD-H column, **3** on the Chiralpak AS-H column, and **4** on the Chiralpak AD-H column were 6.63, 3.48, 7.46, and 8.39, respectively. Most of the separations were obtained by using *n*-Hex/EtOH as the mobile phase. The different elution abilities between EtOH and IPA may be due to the polarity and capability to form hydrogen bonds with CSPs.

It is an important issue to determine the EEO in chiral HPLC separations [46]. As demonstrated in Table 1 and Figure S7, the EEO on polysaccharide-based CSP was reversed (the details shown in Section 2.2) when the type of chiral columns or mobile phase composition were changed, which was consistent with the literature [24,47]. For compound **1**, the EEO changed when EtOH was replaced with IPA as the polar modifier on Chiralpak AD-H column (Figure S7). Similar situation was reported earlier for several chiral sulfoxides, showing that the change in the structure of chiral selector and composition of mobile phase might cause an opposite affinity pattern of enantiomers [16,23,24,39].

### 2.2. AC Assignments of ***1***–***4***

#### 2.2.1. Experimental UV and ECD Spectra

It is significant to directly assign the AC of each peak in the HPLC chromatograms. Without available ECD spectra of enantiopure standard samples, it is essential to establish their AC using a reliable method. In this work, we fell back on ECD spectroscopy together with quantum chemical calculations, which has been widely applied in the AC assignments of chiral organic molecules.

**Figure 3:**
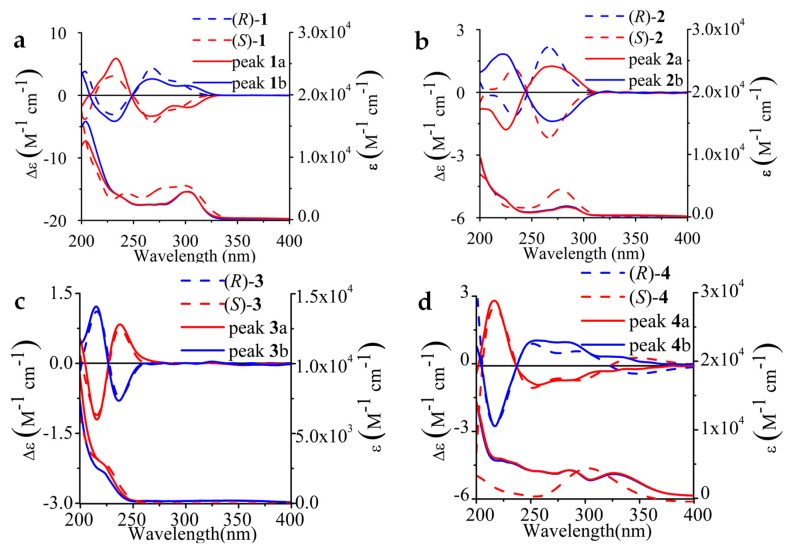
Comparison of experimental and calculated ECD (top) and UV (bottom) spectra of the stereoisomers (**a**–**d**). Solid: experimental in methanol, Dashed: theoretical values, and UV-corrected, bandwidth of **1**–**4** is 0.20 eV, 0.30 eV, 0.30 eV, and 0.40 eV, respectively. The enantiopure isomers of **1**–**4** were prepared under the optimal separation condition of our study, on ChiralPak AD-H, Chiralcel OD-H, ChiralPak AS-H and AD-H columns, respectively. Calculated spectra are Boltzmann averages from calculated spectra of each single conformer.

Experimental ECD and UV spectra of all enantiomers of **1**–**4** in MeOH were obtained (Figure 3). The experimental UV spectra of aromatic alkyl sulfoxides (**1**, **2**) seem to be similar. The UV spectrum of **1** manifests two absorption bands at 205 nm and 300 nm, and that of **2** is slightly blue shifted with an absorption peak at 285 nm, but a shoulder peak at 222 nm. Only one shoulder peak at 225 nm appeared in the UV spectrum of alkyl sulfoxide **3**. For aromatic methyl sulfoxide **4**, there are two absorption bands at 290 nm and 330 nm in its UV spectrum.

It is well known that a pair of enantiomers would share the same UV spectra and have mirrored ECD spectra. Though aromatic alkyl sulfoxides (**1**, **2**, **4**) show the broad peak in the ECD spectra, all these ECD spectra possess an obvious couplet-like feature, which is common in sulfoxide-containing compounds [48]. In the ECD spectra of enantiomeric **1**, peak **1**a presented two negative Cotton effects (CEs) at 300 nm and 268 nm, followed by an intense positive CE at 232 nm, which could be regarded as two branches of a negative, non-degenerate couplet-like feature. Peak **2**a shows a strong positive CE at 270 nm and a negative CE at 222 nm in the ECD spectrum. The ECD spectrum of peak **3**a shows a positive couplet-like feature consisting of two bands, with the first positive CE being at 238 nm and the second negative peak at 216 nm. Nevertheless, peak **4**a gives a wide negative CE signal over a broad range of 235–375 nm, followed by an obvious positive CE peak at 217 nm.

#### 2.2.2. TDDFT Calculation of UV/ECD Spectra

To simulate UV and ECD spectra of **1**–**4**, it is vital to choose suitable combinations of hybrid functional and basis sets [49]. These calculation parameters might greatly affect TDDFT calculated spectra, thus leading to ambiguous AC judgment. Hence, four different hybrid functional/basis set combinations were adopted to verify the consistency of AC assignments. Geometry optimizations and frequency calculations were run at the B3LYP/6-31G(d) and B3LYP/6-31+G(d,p) levels. ECD spectra were predicted employing various combinations of B3LYP and Cam-B3LYP hybrid functionals and 6-31G(d) and 6-311+G(d,p) basis sets. Fortunately, the calculation did give an unambiguous answer to the AC assignment after UV correction if necessary (Figure S8).

Typically, both UV corrections and intensity scaling are applied when the calculated spectra is compared with an experimentally collected one. The best performing calculation method of sulfoxides **1**–**3** was the B3LYP/6-31G(d)//B3LYP/6-31G(d) basis set level, and that of **4** was B3LYP/6-311+G(d,p)//CAM- B3LYP/6-311+G(d,p).

The optimal match with the experimental spectra is shown in Figure 3**.** The first eluted enantiomer of **1** on AD-H, **3** on AS-H and **4** on AD-H were thus assigned as *S*, and **2** on OD-H was assigned as *R*. The agreement between the calculated and experimental ECD spectra of compounds **1**–**3** is almost perfect. The maximum absorption peak of the calculated UV spectra of **4** is at 370 nm, and the experimental UV spectra of **4** shows two absorption bands at 290 nm and 330 nm. In the ECD spectra of enantiomer of **4**, two separate CEs over the range of 240–350 nm are merged into one broad CE.

Moreover, the AC of each peak of **1**–**4** on different chiral columns was also assigned (). The AC judgments of **1**, **2**, and **4** obtained by the TDDFT calculation are consistent with the previously reported results [24,34].

#### 2.2.3. Electron Transitions

Chemical structures of both alkyl sulfoxides **1** and **2** include a substituted pyridyl ring, a benzimidazole ring, and a methyl sulfoxide group. In their ECD spectra, the CEs at 270 nm are associated with two electronic transitions from the sulfoxide chiral center to the benzimidazole ring and the pyridine ring itself. The CE at 230 nm is assignable to π→π* transitions from π-type S=O orbital to the benzene ring and the charge transfer transition in the pyridine ring itself. Compared with **1**, the ECD spectrum of **2** was slightly blue-shifted, which was due to the electron-withdrawing effect of CF_3_ group.

The first CE of alkyl sulfoxide **3** at 238 nm resulted from benzene ^1^La transition, and the second band at 216 nm might be attributed to the sulfinyl group n-π* transition. The oxygen lone pair and π*-type S=O orbitals are heavily mixed with σ and σ*-type S-C orbitals, respectively [50]. The large conjugated aromatic ring leads to a UV spectrum of aromatic methyl sulfoxide **4**, which is distinct from the obtained spectra of the other sulfoxide drugs. The wide absorption band may be assigned to the phenyl ^1^La and ^1^L_b_ transitions. The experimental ECD spectra of **4** appeared a broad peak in the range of 235–375 nm. The second CE of **4** at 220 nm may correspond to the sulfoxide-centered (O=S<) n→π* transition.

### 2.3. Chiral Separation Mechanism and Molecular Docking

The chiral recognition mechanism of polymer-based CSPs is much more complex, because their chiral recognition usually depends on their higher-order structure and the steric fit of the analyte inside the chiral cavity [51,52]. The sulfinyl (>S=O) group, the carbonyl (C=O) group, -OH and -NH groups, the phenyl moiety, benzimidazole, and the pyridyl group in the structure of chiral sulfoxide drugs may form a hydrogen bond, a dipole–dipole bond, π-π interactions and hydrophobic interaction with C=O, -NH- group and the aromatic ring of CSPs. The π-electron density, steric hindrance, various conformations and spatial structures of the polysaccharide glucose unit on CSPs will affect the interaction between the CSPs and chiral molecules [53]. The different types and intensity of the interactions between the sulfoxide compounds and CSPs bring about different chiral separation phenomena. However, recent studies and real understanding of the recognition mechanisms of polysaccharide-based CSPs are far behind their practical applications. 

#### 2.3.1. Common Structural Features

**Figure 4:**
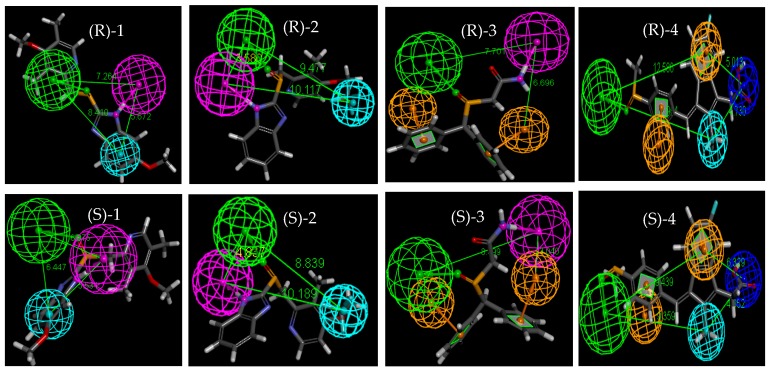
Pharmacophores of **1**–**4** conformers over the most populated cluster. HBA: green, HBD: purple, HY: light blue, AR: orange, NI: mazarine.

During the molecular docking process of **1**–**4**, 100 conformers of each enantiomer were generated and divided into different clusters (Figure S9). The common features of the most populated conformational cluster of **1**–**4** (Figure 4) were created to describe the structural characteristics and better explain the chiral separation mechanisms. There are three types of groups in **1** and **2** which may interact with the CSP, namely, hydrogen bond acceptors (HBA) O=S<, hydrogen bond donors (HBD) -NH-, and hydrophobic moieties (HY) -phenyl/-CF_3_. The features of **3** include HBA O=S<, HBD NH_2_ and aromatic ring (AR) phenyl. The features of **4** are HBA (O=S<), HY (-CH_3_), AR (phenyl) and negative ionizable (NI, -COOH). The apparent difference between the *R* and *S* enantiomer of **1**–**4** is the distance of pharmacophores shown in Figure 4. The differences of distance may influence the stability of the complexes during separation, resulting in different elution of enantiomers.

#### 2.3.2. Molecular Interactions between Analytes and CSPs

The interactions between the enantiomers of **1**–**4** and CSP of the Chiralpak AD-H column are shown in Figure S10. The ligand is depicted as sticks, surrounded by a molecular surface, which is colored according to the interaction with the CSP. Meanwhile, hydrogen bonds are shown as a string of small green spheres. Both enantiomers of **1**–**4** can form one hydrogen bond with the CSP of the Chiralpak AD-H column.

For the enantiomers of **1**, the methoxy group forms a hydrogen bond with an -NH- group of CSP. As for the enantiomers of **2**, one hydrogen bond exists between the O=S< and the amino group of CSP. The carbonyl of **3** interacts with amino moiety of the CSP through a hydrogen bond. There is a hydrogen bond existing between -COOH of **4** and -NH- of the CSP. For **1**, **2**, and **4**, the length of the hydrogen bond of the (*R*)-stereoisomer is shorter than that of the (*S*)-stereoisomer and this is in accordance with the elution sequence on the Chiralpak AD-H column. On the contrary, the length of the hydrogen bond of (*R*)-**3** (2.195 Å) is longer than that of (*S*)-**3** (2.09 Å), and the elution time of (*S*)-**3** is longer on the Chiralpak AD-H column. These results suggest that a shorter hydrogen bond may ensure the stability of the enantiomer in the stationary phase.

#### 2.3.3. Mean Binding Energy

As we know, the enantiomer with lower binding energy could bind more closely to the CSPs and will subsequently be eluted after the other enantiomer. Mean binding energy between the two enantiomers of **1**–**4** and the CSP of the Chiralpak AD-H column are listed in Table 2. The results are concurrent with the elution orders observed in the experiment. 

It is obvious that the enantiomer with a shorter hydrogen bond is more stable than the other enantiomer during the process of elution. Consequently, hydrogen bonding plays a highly important role in the chromatographic separation of sulfoxides on Chiralpak AD-H column. Our result is consistent with high-resolution magic angle spinning nuclear magnetic resonance spectroscopy experiments that the molecular bases for the chiral recognition were the proton interactions between **1** and amylose-based CSP [54]. This also suggests that docking simulations could be adopted to reproduce the elution orders of the enantiomers by the mean binding energy calculations and explain the chiral separation mechanisms visually through the image. Since the mechanisms involved in chiral recognition are complex, a perfect molecular docking method is still a challenging task. We believe our results will aid the development of molecular docking in the application of chiral sulfoxide drugs separation on polysaccharide-based columns.

## 3. Materials and Methods

### 3.1. Materials and Reagents

Sulfoxides **1**–**4** were purchased from National Institutes for Food and Drug Control of China (Beijing, China). All solvents including n-hexane (n-Hex), iso-propyl alcohol (IPA), methanol (MeOH), and ethanol (EtOH) were of HPLC grade. Formic acid (FA) is used as an acidic additive.

### 3.2. Chromatographic Conditions

Compounds **1**–**4** were separated on a Jasco HPLC system consisting of a PU-2089 pump, an AS-2055 sampler, a CO-2060 column thermostat, a MD-2010 detector and a CD-2095 detector. All the chiral columns including Chiralpak AD-H, AS-H, Chiralcel OD-H, and OJ-H (Daicel Chemical Industries, Tokyo, Japan) were 250 × 4.6 mm i.d. with a 5 µm particle size. The detection wavelength of **1**–**4** was set at 275, 275, 240, and 285 nm, respectively. The chromatographic parameters for the enantioselectivity evaluation of the CSPs including retention factor (k), separation factor (α), and resolution factor (Rs) for the enantiomers were calculated.

The samples dissolved in EtOH: n-Hex (80:20, *v*/*v*) were formulated as the solution of 2 mg/mL for analysis and 5 mg/mL for preparation. The enantiopure isomers of **1**–**4** were prepared under the optimal separation condition of our study, on Chiralpak AD-H, Chiralcel OD-H, Chiralpak AS-H and AD-H columns, respectively. Also, the high enantiomeric purities (enantiomeric excesses >99%) of the isolated sulfoxide enantiomers were verified by the enantioselective HPLC system.

### 3.3. ECD Experiments

ECD analysis of the sulfoxide enantiomers was carried out in MeOH on a Jasco J-815 spectrometer. A quartz cuvette with a 1 mm path length was used. The detection wavelength was at 200–400 nm. The spectra were baseline-corrected against MeOH.

### 3.4. Computational Methods

#### 3.4.1. TDDFT Computations

All calculations have been performed on S configuration of **1**–**4**. Preliminary conformational analysis was carried out with the use of the MMFF94 molecular mechanics force field via the MOE software package (Chemical Computing Group, Montreal, QC, Canada) [55]. Geometry optimization and frequency calculation of the MMFF94 conformers were then performed at the B3LYP/6-31G(d) and B3LYP/6-311+G(d,p) levels by using Gaussian 09 (Gaussian, Wallingford, CT, USA) [56]. ECD spectra were predicted employing various combinations of B3LYP or Cam-B3LYP hybrid functionals and 6-31G(d), 6-311+G(d,p) basis sets. All calculations were conducted with the PCM solvation model for MeOH. Calculated UV and ECD spectra of each conformer were simulated at the bandwidth of 0.20–0.40 eV, and the overall spectra were obtained according to the Boltzmann weighting of all conformers. Theoretical ECD and UV spectra were blue-shifted to facilitate the comparison with experimental data if necessary.

#### 3.4.2. Molecular Docking

The 3D-polymer structure of Chiralpak AD-H amylose derivative CSP (AD-12mer.pdb) was downloaded from http://pubs.acs.org [57]. The structure of chiral selector is composed of two AD-12mer.pdb molecules to form “tube-mode” [44]. The structure of AD-H was minimized by means of R2 Dreiding force field using Discovery Studio 2017 software. The structures of the enantiomers of **1**–**4** were minimized in CHARMM force field. AutoDock 4.2.6 (Scripps Research Institute, La Jolla, CA, USA) [58] was adopted to simulate molecular docking. During the docking process, the grid box was set to 30 × 30 × 30 (Å) with 0.375 Å spacing for **1**, **3**, and **4**, 40 × 40 × 40 (Å) with 0.375 Å spacing for **2**.

In consideration of the solvent effect of the mobile phase, the dielectric constant of **1**–**4** was respectively set as 10.668, 5.248, 6.124, and 6.124 based on the weighted average of the mixed mobile phase on the AD-H chiral column. Lamarckian genetic algorithm was used to generate 100 conformations.

## 4. Conclusions

Herein, a systematic study including chromatographic resolution, stereochemical assignment and chiral recognition mechanism of four chiral sulfoxide drugs (**1**–**4**) is conducted. All four compounds have been completely separated on a Chiralpak AD-H column (Figure S11). Among the tested conditions, n-Hex/EtOH as mobile phase is shown to be favorable for the separation of sulfoxides. EEO inversions were observed when the types of chiral columns or mobile phase composition changed. In this work, comparison of the ECD spectra with the TDDFT calculated data provided a robust way to assign the AC of their stereoisomers. Moreover, the observed EEOs were found to be in accord with mean binding energy calculations. The docking simulation could also explain visually the underlying chiral separation mechanisms. This work has the potential to build up an experimental and theoretical methodology to facilitate the stereochemistry and chiral recognition mechanism of chiral drugs.

## Figures and Tables

**Table 1:** Comparison of sulfoxide drugs on chiral columns.

CSP	Compound	Mobile Phase (*v*/*v*)	t_R1_ (min)	t_R2_ (min)	k_1_	k_2_	α	Rs	Sign ^e^
AD-H	**1**	*n*-Hex:EtOH (60:40) ^a,c^	12.97	20.26	3.32	5.80	1.75	6.63	(*S*)-(−)
	**2**	*n*-Hex:IPA (80:20) ^a,d^	18.89	21.04	5.30	6.01	1.13	2.02	(*S*)-(−)
	**3**	*n*-Hex:EtOH (80:20) ^a,c^	19.88	26.00	2.98	4.20	1.45	5.85	(*R*)-(−)
	**4**	*n*-Hex:FA:EtOH (80:0.1:20) ^a,c^	14.96	22.87	1.99	3.57	1.79	8.39	(*S*)-(−)
AS-H	**1**	*n*-Hex:EtOH (60:40) ^b,c^	8.11	12.45	1.70	3.15	1.85	5.54	(*R*)-(+)
	**3**	*n*-Hex:EtOH (60:40) ^b,d^	13.64	21.83	1.73	3.37	1.95	7.46	(*S*)-(+)
OD-H	**1**	*n*-Hex:EtOH (90:10) ^a,d^	18.16	22.40	5.05	6.47	1.28	2.54	(*S*)-(−)
	**2**	*n*-Hex:IPA (80:20) ^a,c^	18.48	27.43	5.16	8.14	1.58	3.48	(*R*)-(+)
OJ-H	**2**	*n*-Hex:EtOH (95:5) ^a,d^	33.95	36.68	10.31	11.22	1.09	1.67	(*R*)-(+)
	**3**	*n*-Hex:EtOH (55:45) ^b,d^	16.06	21.01	2.21	3.20	1.46	5.43	(*S*)-(+)

Retention factor k = (t_1_ − t_0_)/t_0_, Resolution factor Rs = 2(t_2_ − t_1_)/(w_1_ + w_2_). t_1_, t_2_ is retention time of enantiomer. t_0_ is dead time. w_1_, w_2_ is peak width of enantiomer. Selectivity factor α = k_2_/k_1_ = (t_2_ − t_0_)/(t_1_ − t_0_)]. Detection wavelengths of **1**–**4** are 275, 275, 240, and 285 nm, respectively. ^a^ Flow rate: 1 mL/min. ^b^ Flow rate: 0.8 mL/min. ^c^ Column temperature: 30 °C. ^d^ Column temperature: 25 °C. ^e^ Sign of the absolute configuration of the first eluted enantiomer.

**Table 2:** The calculated average binding energies for compounds **1**–**4** on the ChiralPak AD-H column.

Entry	Mean Binding Energy (kcal/mol)	The Length of Hydrogen Bond (Å)	Elution Time (min)	Rs
(*R*)-**1**	−5.87	1.932	20.26	6.63
(*S*)-**1**	−5.43	2.003	12.97	
(*R*)-**2**	−6.71	2.054	21.04	2.02
(*S*)-**2**	−6.01	2.232	18.89	
(*R*)-**3**	−7.19	2.195	19.88	5.85
(*S*)-**3**	−7.82	2.090	26.00	
(*R*)-**4**	−7.92	1.762	22.87	8.39
(*S*)-**4**	−6.52	2.016	14.96	

## Supplementary Materials

The following materials are available online. (1) Figure S1. Plots showing resolution factors of the enantiomers of **1**–**3** as a function of the n-Hex content in the mobile phase (A), temperature (B) and flow rate (C). (2) Table S1. Effect of the acidic additive on the resolution of **4** on the AD-H column. (3) Figure S2. Comparison of the UV (upper) and ECD (lower) chromatograms of **4** on ChiralPak AD-H column with acidic additive. (4) Figure S3. The UV (upper) and ECD (lower) chromatograms of **1** on ChiralPak AD-H column under the optimal condition. (5) Figure S4. The UV (upper) and ECD (lower) chromatograms of **2** on Chiralcel OD-H column under the optimal condition. (6) Figure S5. The UV (upper) and ECD (lower) chromatograms of **3** on ChiralPak AS-H column under the optimal condition. (7) Figure S6. The UV (upper) and ECD (lower) chromatograms of **4** on ChiralPak AD-H column under the optimal condition. (8) Figure S7. Comparison of the UV (upper) and ECD (lower) chromatograms of **1** on ChiralPak AD-H column with EtOH and IPA. (9) Figure S8. Conformational distribution of enantiomers **1**–**4** during the docking process. (10) Figure S9. Comparison of TDDFT-calculated ECD and UV spectra. (11) Figure S10. Interactions between two enantiomers of **1**–**4** and the CSP of the AD-H column. (12) Figure S11. Graphic illustrating the resolution of chiral sulfoxides on the chiral columns.
