# Supplementary material for: Enantioseparation, Stereochemical Assignment and Chiral Recognition Mechanism of Sulfoxide-Containing Drugs
Source: Molecules. 2018 Oct 18;23(10):2680. doi: 10.3390/molecules23102680 (PMC6222453; doi:10.3390/molecules23102680)
Supplement: Supplementary file 1 [file molecules-23-02680-s001.pdf]

Supplementary data

Enantioseparation, stereochemical assignment and chiral recognition mechanism of sulfoxide-containing drugs

Fei Xiong, Bei-Bei Yang, Jie Zhang and Li Li*

Beijing Key Laboratory of Active Substances Discovery and Druggability Evaluation, Institute of Materia Medica, Chinese Academy of Medical Sciences & Peking Union Medical College, Beijing 100050, P.R. China.

* Correspondence: annaleelin@imm.ac.cn; Tel.: +86-10-63165247

The List of Contents

**No.**	**Content**	**Page**
1	**Figure S1.** Plots showing resolution factors of the enantiomers of **1-3** as a function of the *n*-Hex content in the mobile phase (A), temperature (B) and flow rate (C).	S3
2	**Table S1.** Effect of the acidic additive on the resolution of **4** on the AD-H column.	S4
3	**Figure S2.** Comparison of the UV (upper) and ECD (lower) chromatograms of **4** on ChiralPak AD-H column with acidic additive.	S5
4	**Figure S3.** The UV (upper) and ECD (lower) chromatograms of **1** on ChiralPak AD-H column under the optimal condition.	S6
5	**Figure S4.** The UV (upper) and ECD (lower) chromatograms of **2** on Chiralcel OD-H column under the optimal condition.	S7
6	**Figure S5.** The UV (upper) and ECD (lower) chromatograms of **3** on ChiralPak AS-H column under the optimal condition.	S8
7	**Figure S6.** The UV (upper) and ECD (lower) chromatograms of **4** on ChiralPak AD-H column under the optimal condition.	S9
8	**Figure S7.** Comparison of the UV (upper) and ECD (lower) chromatograms of **1** on ChiralPak AD-H column with EtOH and IPA.	S10
9	**Figure S8.** Conformational distribution of enantiomers **1-4** during the docking process.	S11
10	**Figure S9.** Comparison of TDDFT-calculated ECD and UV spctra.	S12
11	**Figure S10.** Interactions between two enantiomers of **1-4** and the CSP of the AD-H column.	S13
12	**Figure S11.** Graphic illustrating the resolution of chiral sulfoxides on the chiral columns.	S14

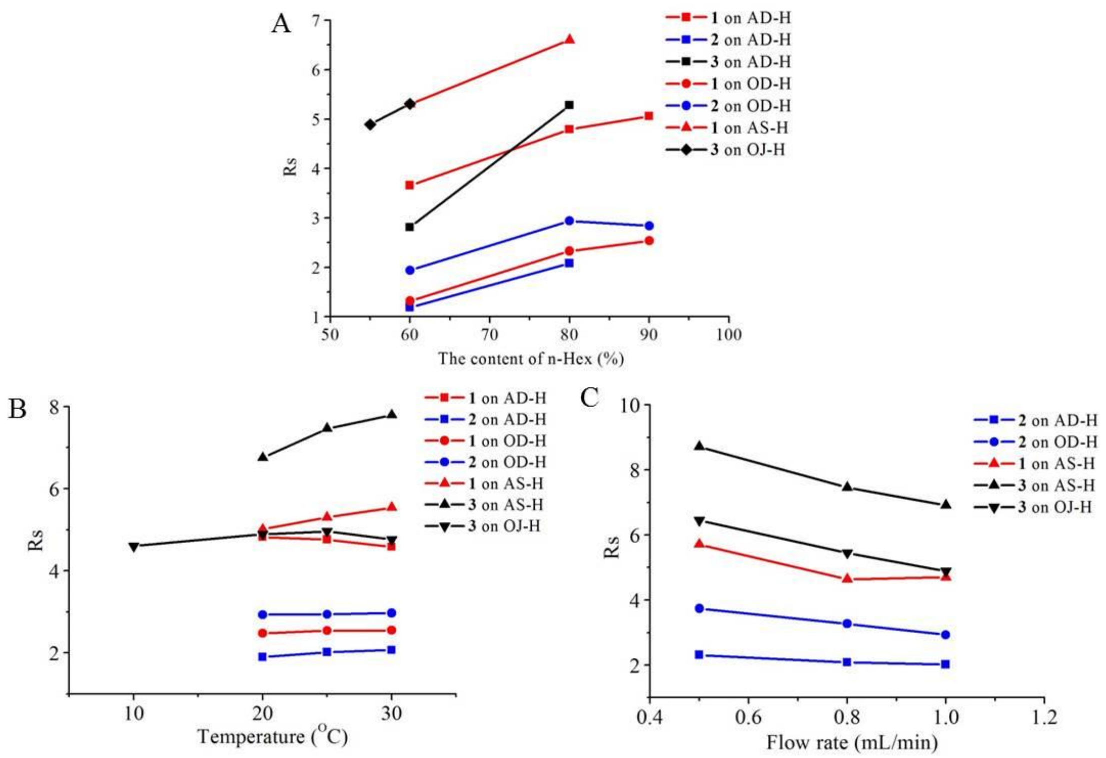

Figure S1. Plots showing resolution factors of the enantiomers of **1**-**3** as a function of the *n*-*H*ex content in the mobile phase (A); temperature (B) and flow rate (C)**.**

Table S1. Effect of the acidic additive on the resolution of **4** on the AD-H column.

Mobile phase	t_{R1} (min)	t_{R2} (min)	k_1	k_2	α	Rs
n-Hex:EtOH (80:20, v/v)[a]	12.63	15.96	1.53	2.19	1.44	2.19
n-Hex:FA:EtOH (80:0.1:20, v/v/v)[b]	14.96	22.87	1.99	3.57	1.79	8.39

[Retention factor $k = (t_1 - t_0)/t_0$, Resolution factor $Rs = 2(t_2 - t_1)/(w_1 + w_2)$. t_1, t_2 is retention time of enantiomer. t_0 is dead time. w_1, w_2 is peak width of enantiomer. Selectivity factor $\alpha = k_2/k_1 = (t_2 - t_0)/(t_1 - t_0)$]. Flow rate: 1 mL/min; column temperature: 30°C; [a] Detection wavelength: 235 nm; [b] Detection wavelength: 285nm.

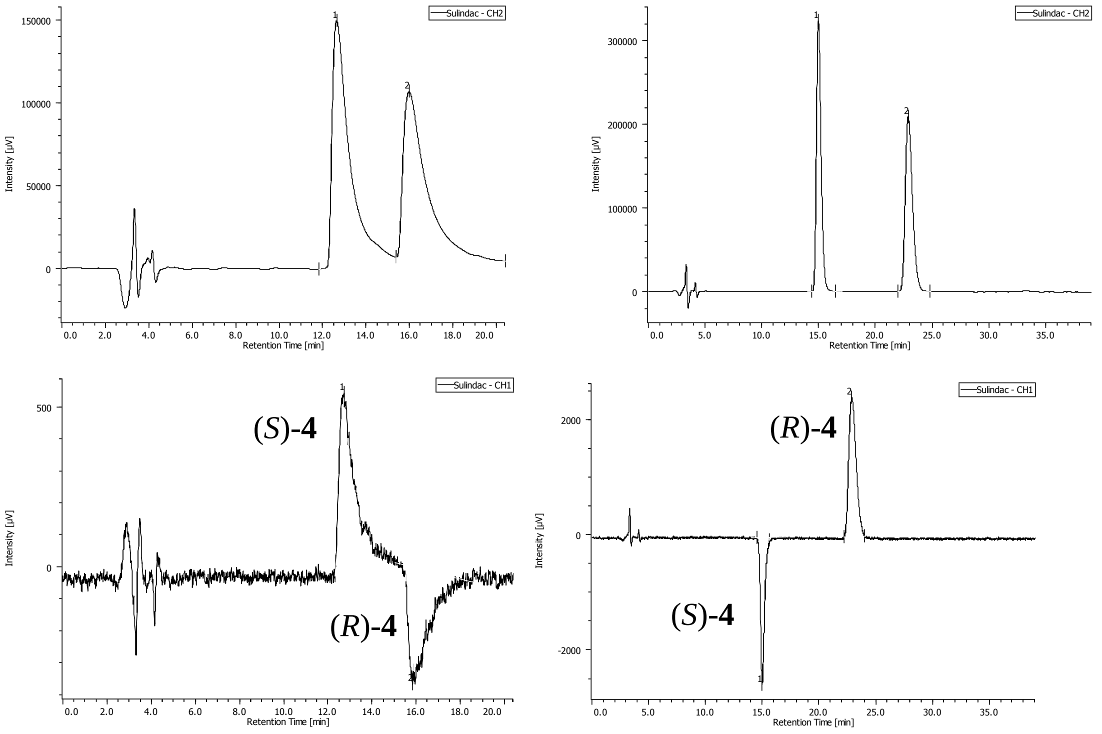

Figure S2. Comparison of the UV (upper) and ECD (lower) chromatograms of **4** on ChiralPak AD-H column with acidic additive, flow rate is 1.0 mL/min, and column temperature is 30°C. Detection wavelength of **4** on the left is 235 nm, on the right is 285 nm. Mobile phase of **4** on the left is n-Hex: EtOH (80: 20, v/v), on the right is n-Hex: FA: EtOH (80: 0.1: 20, v/ v/v).

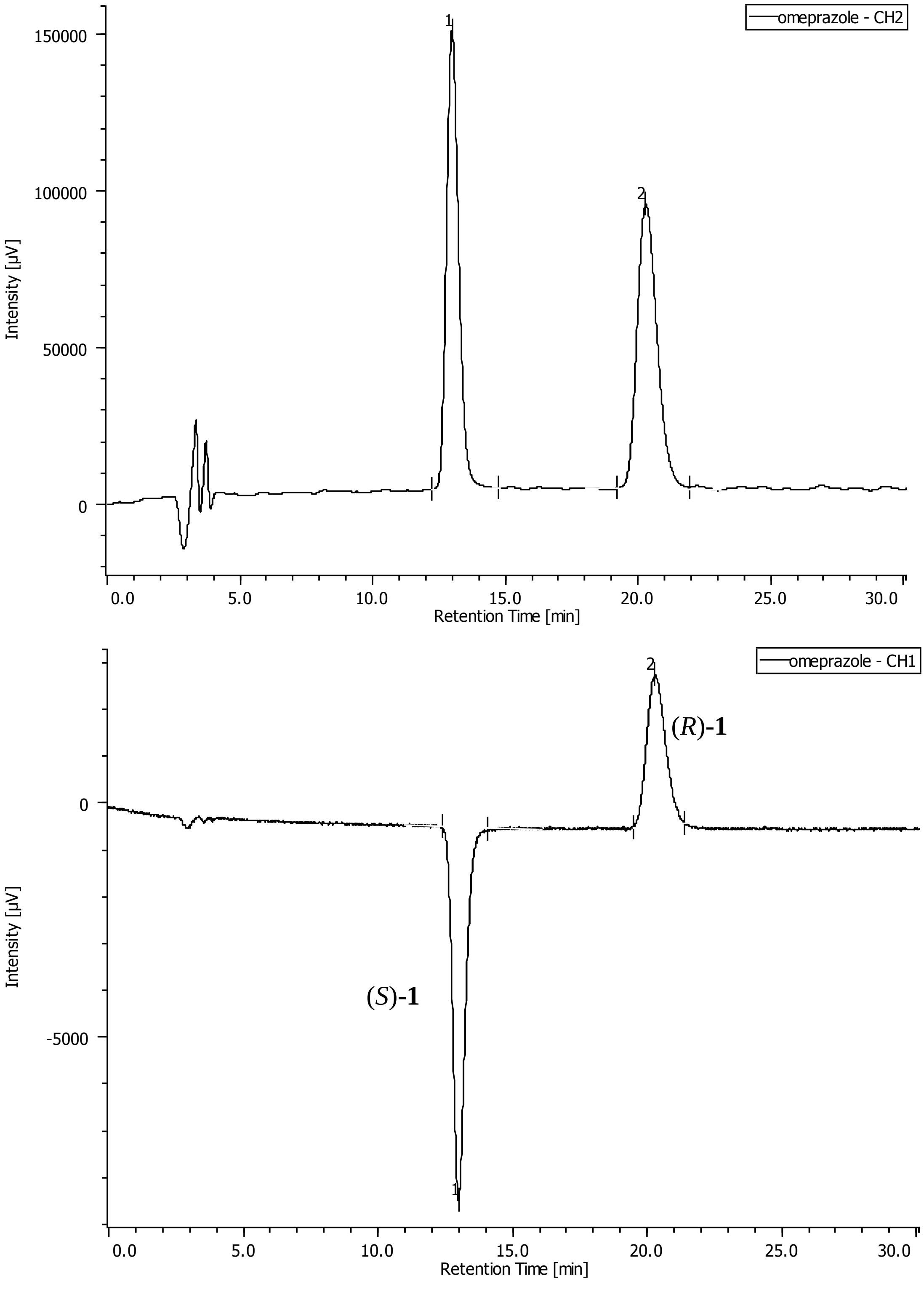

Figure S3. The UV (upper) and ECD (lower) chromatograms of **1** on ChiralPak AD-H column under the optimal condition. Mobile phase is *n*-Hex:EtOH 60:40, flow rate is 1.0 mL/min, column temperature is 30°C and detection wavelength is 275 nm.

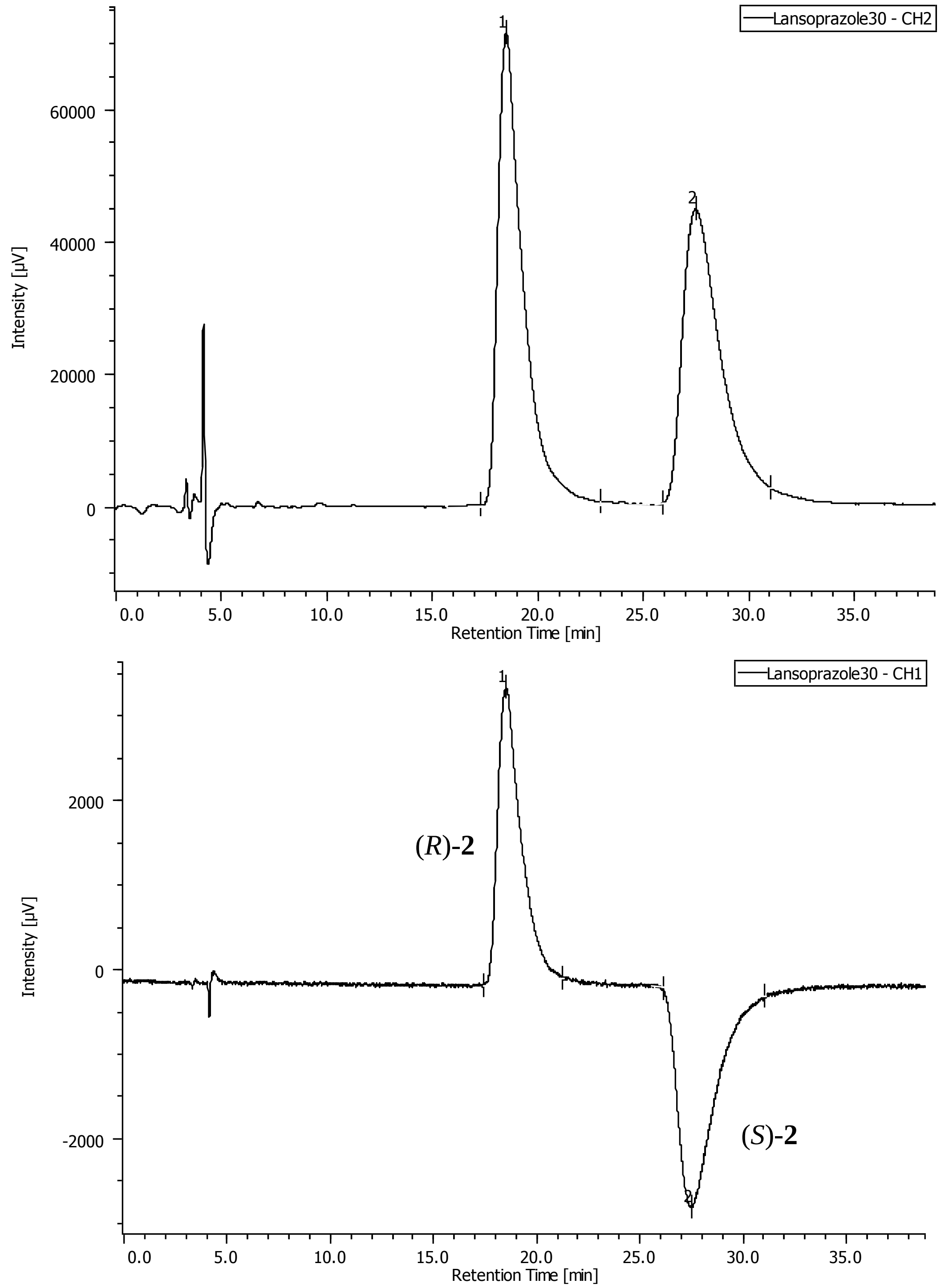

Figure S4. The UV (upper) and ECD (lower) chromatograms of **2** on Chiralcel OD-H column under the optimal condition. Mobile phase is *n*-Hex:EtOH 90:10, flow rate is 1.0 mL/min, column temperature is 25°C and detection wavelength is 275 nm.

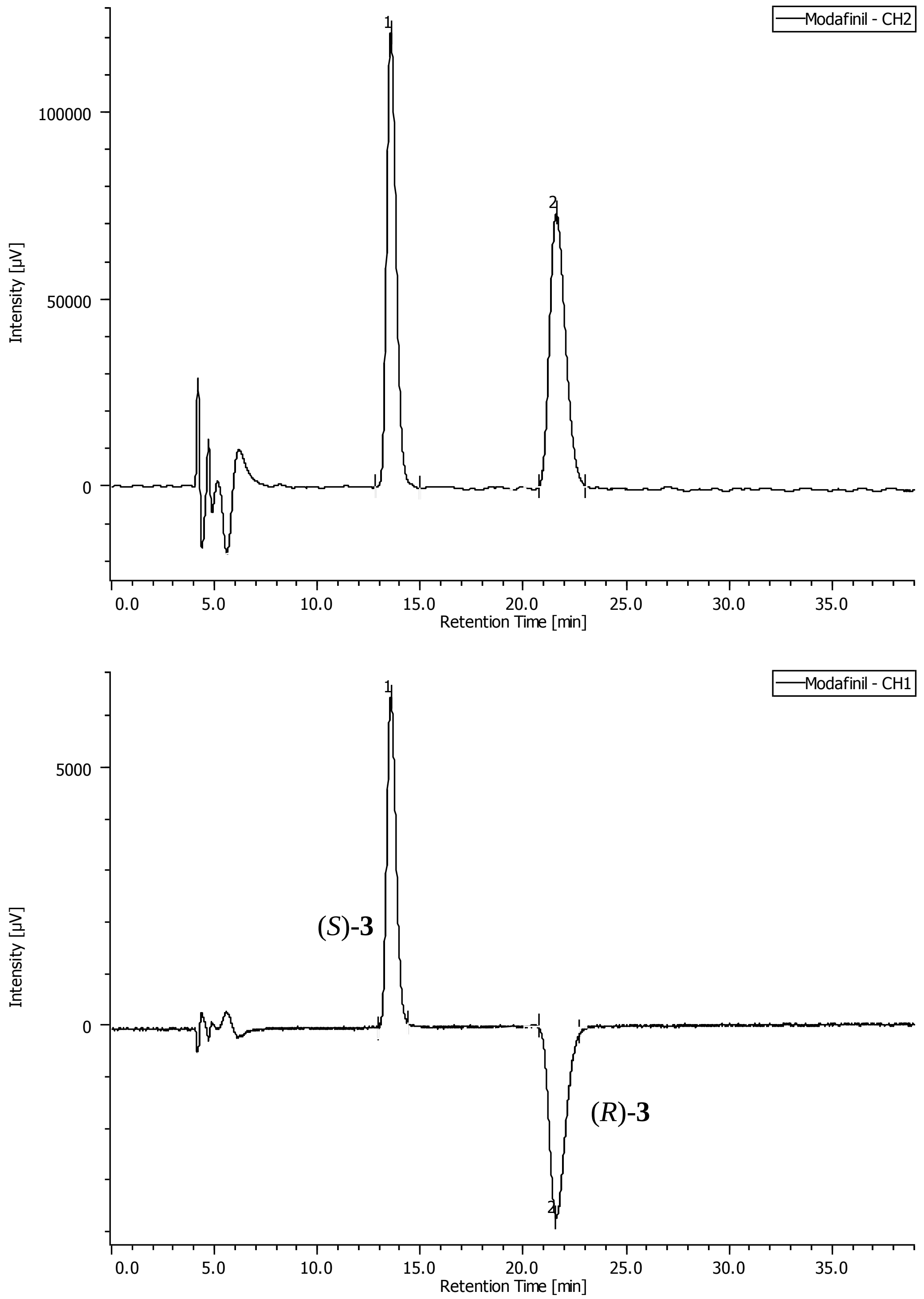

Figure S5. The UV (upper) and ECD (lower) chromatograms of **3** on ChiralPak AS-H column under the optimal condition. Mobile phase is *n*-Hex:EtOH 60:40, flow rate is 0.8 mL/min, column temperature is 25°C and detection wavelength is 240 nm.

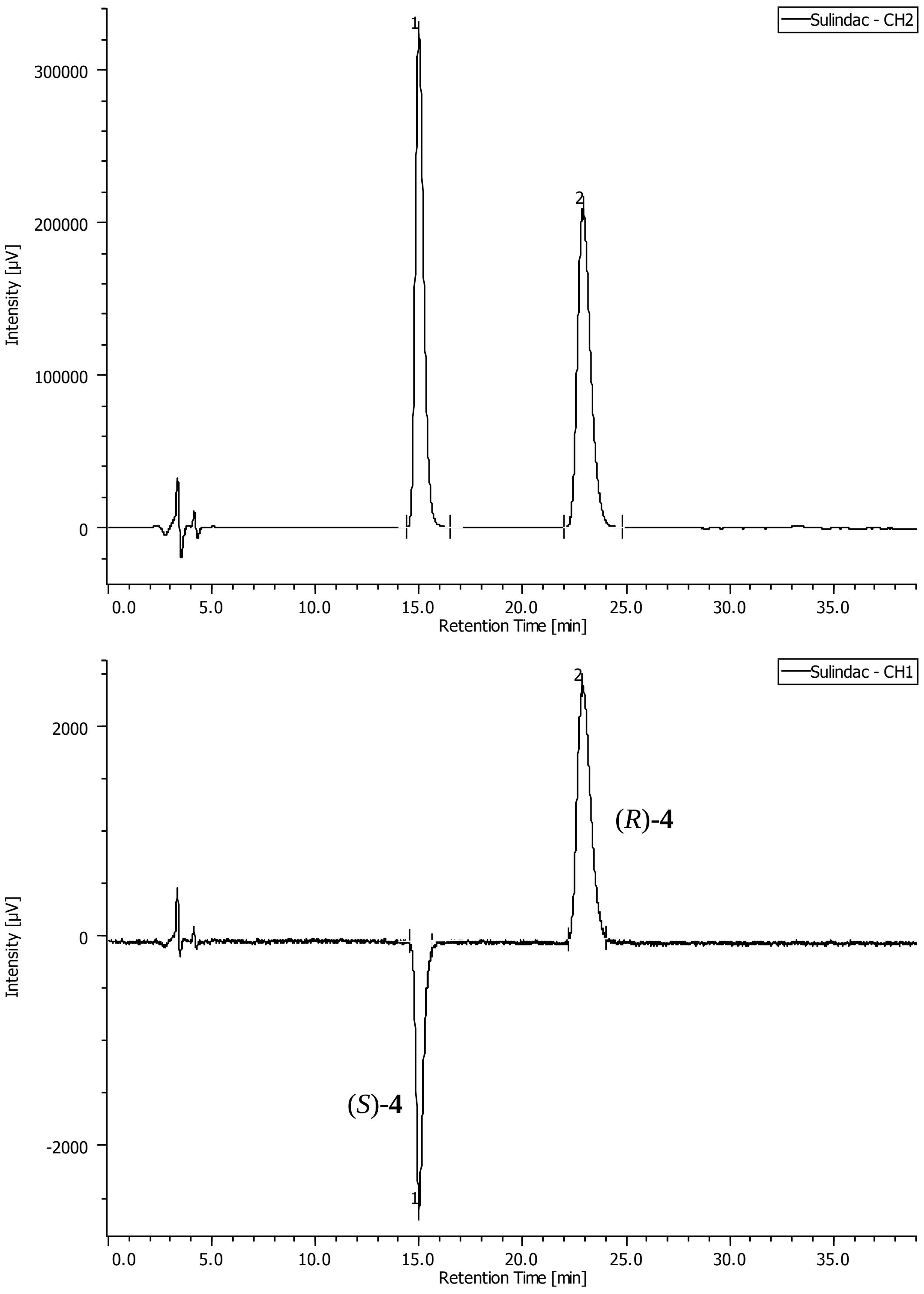

Figure S6. The UV (upper) and ECD (lower) chromatograms of **4** on ChiralPak AD-H column under the optimal condition. Mobile phase is n-Hex: FA: EtOH (80: 0.1: 20, v/ v/v), flow rate is 1.0 mL/min, column temperature is 30°C and detection wavelength is 285 nm.

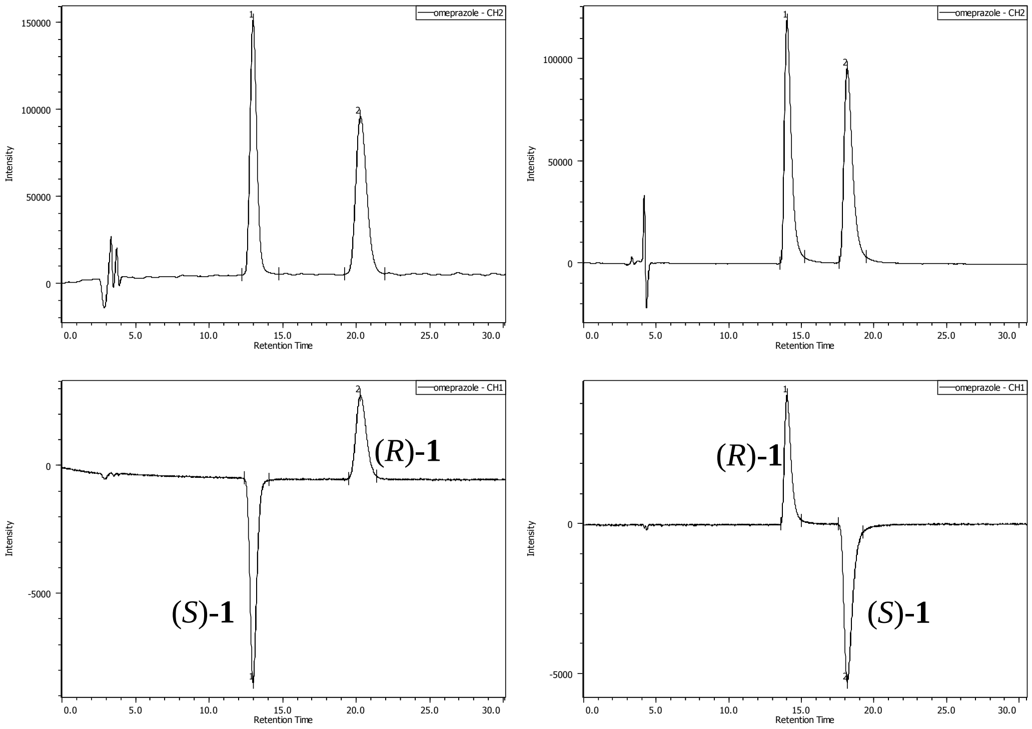

Figure S7. Comparison of the UV (upper) and ECD (lower) chromatograms of **1** on ChiralPak AD-H column with EtOH 40and IPA20, flow rate is 1.0 mL/min, and column temperature is 30°C and detection wavelength is 275 nm.

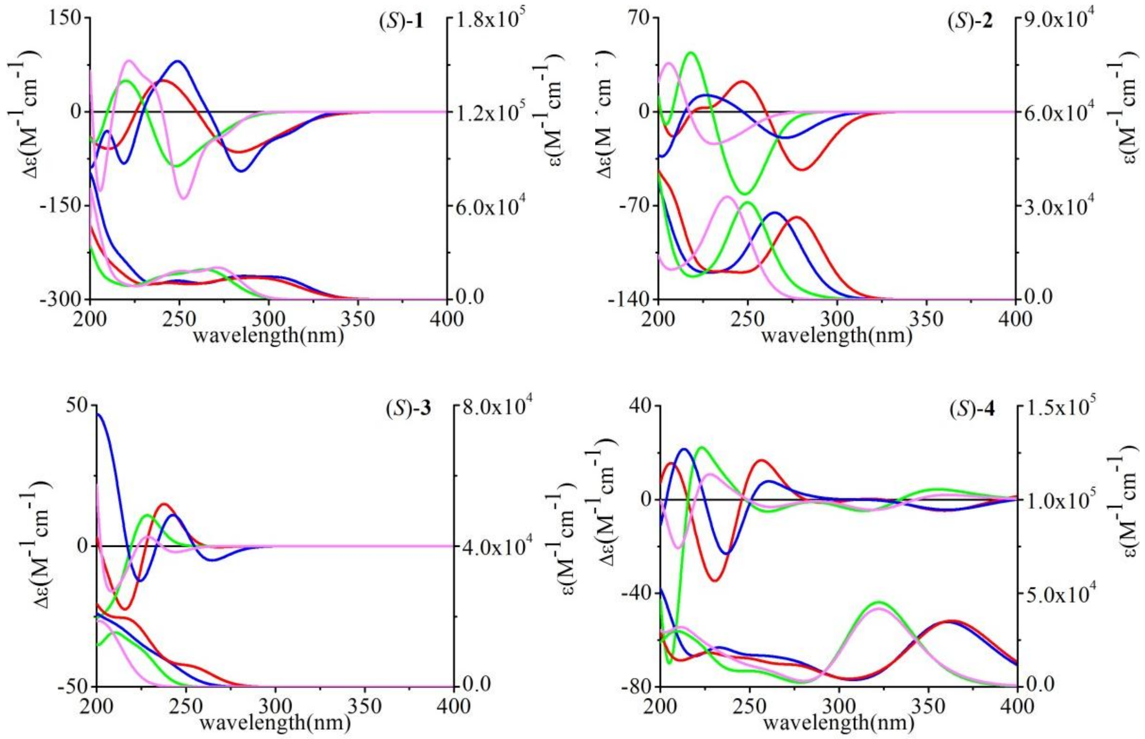

Figure S8. Comparison of TDDFT-calculated ECD and UV spectra (top and bottom respectively) for sulfoxides **1-4**, theoretical, B3LYP/6-31G(d)// B3LYP/6-31G(d) (red); CAM-B3LYP/6-31G(d)//B3LYP/6-31G(d) (green); B3LYP/6-311+G(d,p)//B3LYP/6-311+G(d,p) (blue); CAM-B3LYP/6-311+G(d, p)//B3LYP/6-311+G(d,p) (pink). Calculated spectra are Boltzmann averages from calculated spectra of each conformer.

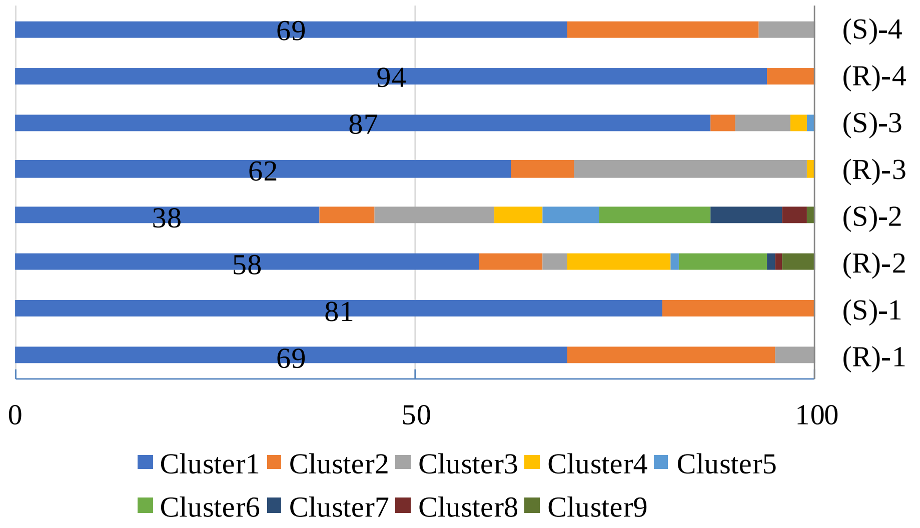

Figure S9. Conformational distribution of enantiomers of **1-4** during the docking process.

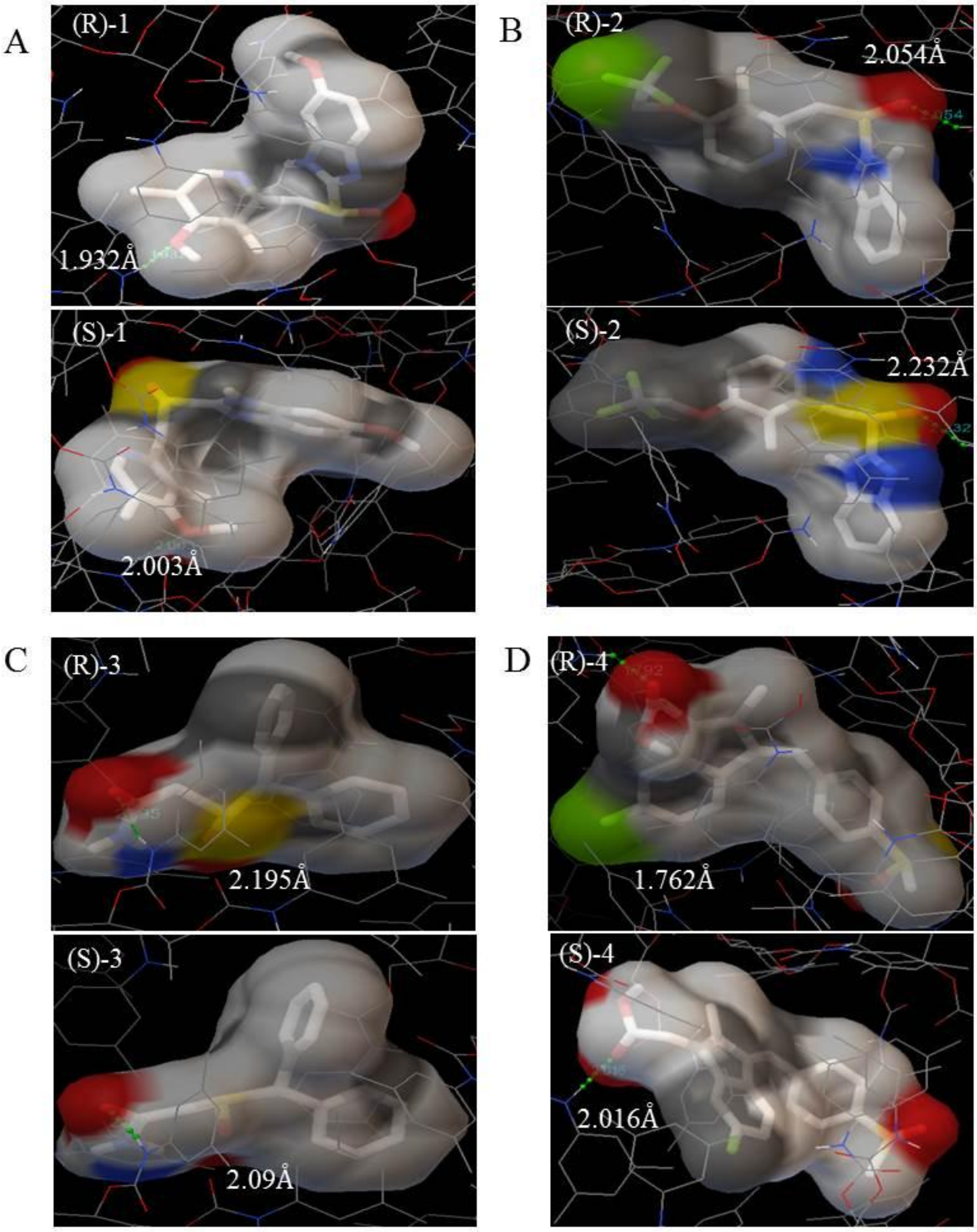

Figure S10. Interactions between two enantiomers of **1-4** and the CSP of the AD-H column. The conformers shown of molecules **1-4** is the lowest binding energy in their most populated cluster. The structure of CSP is composed of two AD-12mer.pdb molecules to form "tube-mode" [44].

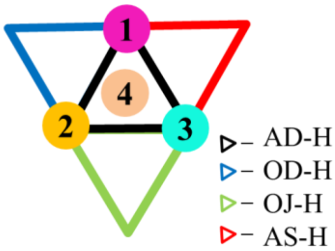

Figure S11. Graphic illustrating the resolution of chiral sulfoxides on the chiral columns.
